# Effects of anesthesia with sevoflurane on outcome parameters in murine experimental studies

**DOI:** 10.1007/s00068-024-02583-y

**Published:** 2024-07-09

**Authors:** Jonas C. Wrba, Ludmila Lupu, Sonja Braumüller, Thomas A. Neff, Rebecca Halbgebauer, Annette Palmer, Markus Huber-Lang

**Affiliations:** 1 Institute of Clinical and Experimental Trauma Immunology, Ulm University Medical Center, University Hospital of Ulm, Helmholtzstr. 8/1, 89081 Ulm, Germany; 2 Department of Trauma, Orthopedic, Plastic and Hand Surgery, University Hospital of Augsburg, Augsburg, Germany; 3 Department of Anaesthesia and Intensive Care Medicine, Cantonal Hospital of Muensterlingen, Münsterlingen, Switzerland

**Keywords:** Murine trauma model, Anesthesia, Inflammatory response, Organ dysfunction

## Abstract

**Purpose:**

Multiple murine studies modelling the immuno-pathophysiological consequences of trauma, shock, burn or sepsis were performed during the last decades. Almost every animal model requires anesthesia for practical and ethical reasons. Furthermore, often, corresponding control groups involve untreated animals without or with a limited exposure to anesthetics. However, the influences of anesthetic drugs on immuno-pathophysiological reactions remain insufficiently investigated. Therefore, we aimed to closer characterize the anesthetic impact exemplified by sevoflurane on the organ performance in mice and thereby investigate the influence of anesthesia itself on major outcome parameters in animal studies.

**Methods:**

C57/BL6 mice were subjected either to 270 min of sevoflurane narcosis or directly euthanized. Plasma, BAL-fluids, lungs, kidneys, liver and intestine were collected and examined for immunological, functional and morphological changes.

**Results:**

Systemic levels of the cytokine keratinocyte chemoattractant (KC) were raised in the narcosis group, while concentrations of high mobility group box protein 1 (HMGB-1) as a major inflammatory marker were reduced. In the lungs, levels of HMGB-1 and interleukin 6 (IL-6) were reduced. In contrast, systemic concentrations of intestinal fatty acid binding-protein (i-FABP) as an intestinal damage marker were elevated. Furthermore, liver-type fatty acid binding-protein (L-FABP) levels were lower in the narcosis animals, and inflammatory markers were reduced in liver tissues. Anesthesia also ameliorated the inflammatory reaction in renal tissues, while plasma levels of urea and creatinine were elevated, reflecting either dehydration and/or impaired renal function.

**Conclusion:**

As anesthesia with sevoflurane exhibited distinct effects in different organs, it is difficult to predict its specific impact on targets of interest in in vivo studies. Therefore, further studies are required to clarify the effects of different anesthetic drugs. Overall, the inclusion of a control group subjected to the same anesthesia protocol as the experimental groups of interest seems helpful to precisely define the inherent impact of the anesthetic when investigating immuno-pathophysiologic conditions in vivo.

## Introduction

During the last decades, numerous animal studies were performed to examine how various clinically relevant pathologic conditions, such as trauma, shock or sepsis, would alter the immuno-pathophysiologic reaction on a multi-organ level. These animal studies are considered relevant to gain further insights into molecular mechanisms of the complex underlying immuno-pathophysiology [1–3]. Undoubtedly, most study protocols in animal trauma research require any form of sufficient narcosis for ethical, technical and scientific reasons. However, it often remains unclear, which alterations of the immunological and organ functions are caused by the trauma impact itself, and which by the narcosis applied. Different narcotics and analgesic drugs have been investigated in a variety of studies to clarify their effects on the immune system [4]. Various effects have been discovered for different classes of drugs, and sometimes even within the same class. Volatile anesthetics seem to exhibit rather anti-inflammatory effects regarding both innate and adaptive immunity [5–12]. In contrast, the most commonly used intravenous anesthetic propofol appears to exert less modulatory effects on the innate immune system [11, 12]. Opioids are most frequently used for analgesia in experimental studies. Several immunosuppressive effects, such as reduction of the phagocytic activity, production of reactive oxygen species (ROS) and cytokines have been shown in different study settings, with remarkable differences between several specific opioids [13, 14]. The data mentioned above originate from a wide range of different study protocols, and mostly was generated in vitro using cell lines or blood samples from human patients. Surprisingly, there exist only few published data on effects of narcosis in small animal models. Thus, it is difficult to specify the inherent impact of anesthetics in animal studies although their application is mandatory in trauma-, shock-, and critical care research. We therefore aim to investigate in this exemplary descriptive study, which acute alterations on the immune system and organ performance become evident in a standard murine model by a sevoflurane narcosis per se in the absence of any additional trauma or other pathologic condition.

## Materials and methods

### Study protocol

Animal experiments were performed according to the National Institutes of Health Guidelines for the use of laboratory animals. The study protocol was approved by the University Animal Care Committee and the Federal Authorities for animal research, Tuebingen, Germany (Approval No. 1194).

8–12 weeks old male C57BL/6 mice were randomly assigned to either the narcosis or the control group (ctrl). 6 animals per group were enrolled. Mice in the narcosis group were consistently kept under sevoflurane anesthesia (Sevorane^®^ Abbott, Wiesbaden, Germany) for 270 min following a standardized protocol. Mice were spontaneously breathing through a tightly fitted ventilation mask, avoiding any mechanical ventilatory support. They were placed on a feedback heating pad with rectal temperature measurement and kept under a piece of tinfoil to maintain physiological body temperature. After the end of the experimental period, under narcosis with 5% sevoflurane, mice were sacrificed by exsanguination via cardiac puncture and bilateral pneumothorax. Animals of the control group were sacrificed similarly but without the previous 270 min sevoflurane exposure. After sacrifice, blood was withdrawn by cardiac puncture and tissue samples from lungs, liver, kidneys, and the intestine were collected for further analysis.

### Preparation of bronchoalveolar lavage samples

For the collection of the bronchoalveolar lavage (BAL), the trachea was dissected and cannulated, the left lung was clamped and the right lung was flushed 3 times with 0.5 ml PBS containing a 1:1000 broad spectrum protease inhibitor (Sigma-Aldrich, St. Louis, MO, USA). Afterwards, BAL fluids were centrifuged (10 min at 450 g and 4 °C) and the supernatant was stored at -80 °C until analysis. The cell pellet was resuspended in 100 µl PBS. 10 µl of this suspension were mixed with crystal violet to determine the total cell number in a Neubauer chamber by light microscopy. The residual cell suspension was subjected to cytospin preparation (Shandon Cytospin 3, Thermo Scientific, Dreieich, Germany). Cytospins were fixed and stained with Hemacolor rapid stainig kit (Merck, Darmstadt, Germany) and 300 cells for each experimental condition were differentially analyzed by light microscopy to determine the ratio of infiltrated neutrophils and mononuclear cells in the BAL.

### ELISA

Measurement of cytokines in the plasma of mice was performed by sandwich enzyme-linked immunosorbent assay (ELISA). The following ELISA kits were used according to the manufacturers’ instructions: Mouse IL-6 ELISA Kit (BD Sciences, Franklin Lakes, USA); Mouse KC ELISA Kit (R&D Systems, Minneapolis, USA); Mouse HMGBI ELISA Kit (IBL, Hamburg, Germany); Mouse CC16 ELISA Kit (Abbexxa, Cambridge, UK); Mouse I-FABP ELISA Kit (LSBio Seattle, USA); Mouse L-FABP ELISA Kit (LSBio Seattle, USA).

### Statistical analyses

Statistical analyses were performed with the help of GraphPad Prism software (Edition 9.1.2). We assumed normal distribution. The results are displayed as mean ± standard deviation (SD). For elimination of outliers we applied the Z-score test. For comparison of differences Student’s t test was used. The significance level was defined as *p* < 0.05 and was asterisked: *p < 0.05, **p < 0.01, ***p < 0.001.

## Results

### Systemic effects

**Fig. 1:**
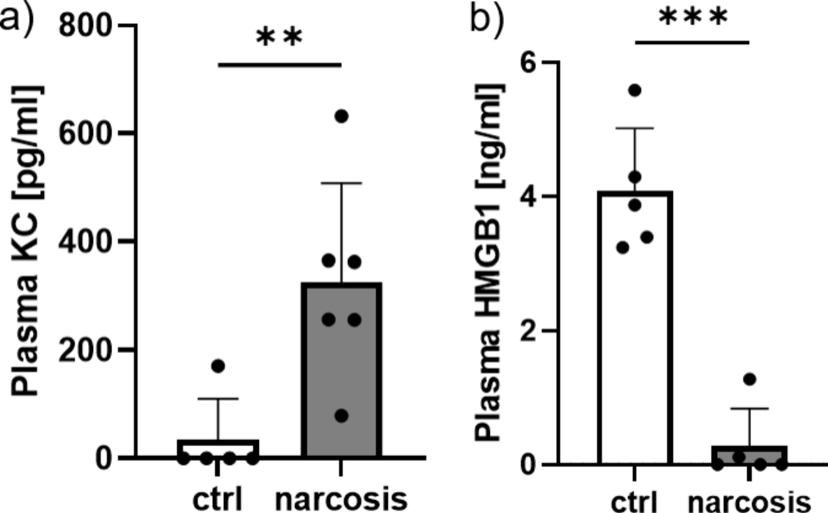
Influence of sevoflurane narcosis on systemic inflammation. Plasma concentrations of KC were significantly elevated in the narcosis group compared to control (ctrl) animals (**a**), while plasma levels of high mobility group box 1 protein (HMGB1) were reduced (**c**). *n* = 5–6 per group, **p < 0.01, ***p < 0.001, t-test, mean ± SD, 1 outlier removed from **b**) narcosis group

Plasma levels of KC (the murine homologous of human IL-8) were significantly elevated in animals exposed to 270 min narcosis as compared to the control group (Fig. 1a). IL-6 was not detectable in the plasma of any group, while plasma concentrations of HMGB-1 were reduced in the narcosis group (Fig. 1b).

### Effects of anesthesia on the lungs

**Fig. 2:**
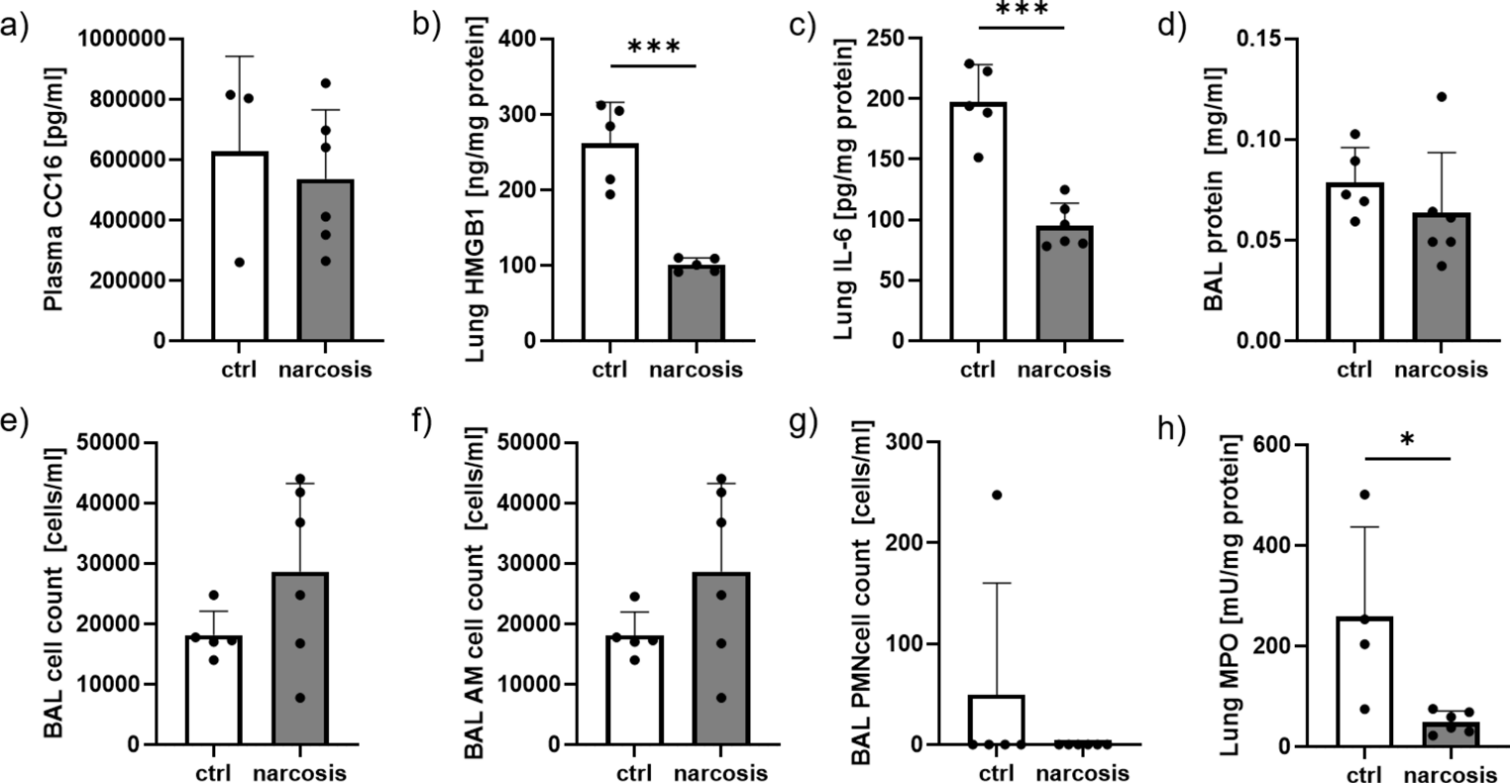
Effects of narcosis on the lungs. Plasma levels of club cell secretory protein 16 (CC16) remained unaltered between both groups (**a**). In bronchoalveolar lavage fluid (BAL), total protein concentration remained unchanged (**d**), while levels of high mobility group box protein 1 (HMGB1, **b**) and interleukin 6 (IL-6, **c**) were reduced in the experimental group. The total cell count (**e**) and the number of alveolar macrophages (AM, **f**) were tendentially higher in the experimental group, while the amount of polymorphonuclear cells seemed to be reduced after narcosis (**g**). Activity of myeloperoxidase (MPO) in lung tissue was significantly reduced in the narcosis group (**h**). *n* = 3–6 per group, *p < 0.05, ***p < 0.001, t-test, mean ± SD, no outliers

Plasma levels of Club cell secretory protein 16 (CC-16) as a systemic marker for lung damage were not altered by the narcosis protocol performed in this study (Fig. 2a). Tissue levels of HMGB-1 (Fig. 2b) and IL-6 (Fig. 2c) were reduced in lung tissues of animals subjected to sevoflurane. Total protein concentration in BAL fluids, an indicator for blood-air-barrier disruption, was similar in both groups (Fig. 2d), while for total cell counts (Fig. 2e) and amounts of alveolar macrophages (AM), (Fig. 2f) there was a trend towards higher levels in samples of animals subjected to sevoflurane narcosis. In contrast, polymorphonuclear cells (PMN) were reduced (Fig. 2g; not significant), which also was reflected by significantly lower MPO concentrations in the BAL fluids of anesthetized animals in comparison to control mice (Fig. 2h).

### Changes in intestine and liver

**Fig. 3:**
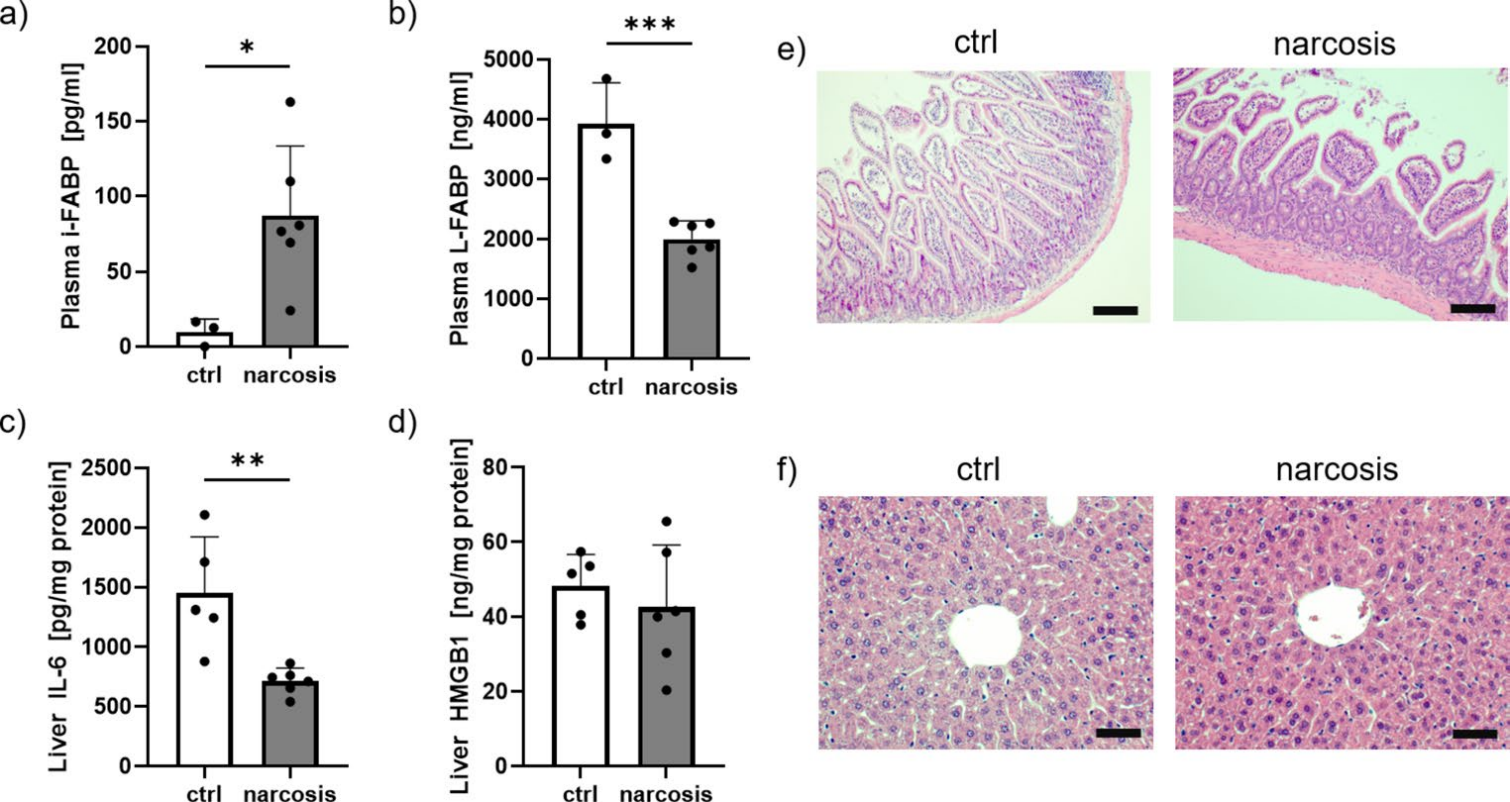
Alterations in intestine and liver. Plasma levels of intestinal fatty-acid binding protein (i-FABP, **a**) were significantly elevated after narcosis, while levels of liver-type fatty-acid binding protein (L-FABP, **b**) were lowered. Concentrations of interleukin 6 (IL-6) in liver tissue were also reduced (**c**), while levels of high mobility group box protein 1 (HMGB1) remained unchanged (**d**). In histological examination, there was no visible difference between animals of the control and the narcosis group neither in the intestine (**e**), nor in the liver (**f**). Scale bars indicate 100 μm in (**e**) and 50 μm in (**f**). *n* = 3–6 per group, *p < 0.05, **p < 0.01, ***p < 0.001, t-test, mean ± SD, no outliers

The plasma concentrations of intestinal fatty acid-binding protein (i-FABP) were significantly higher in the narcosis group than in the control group, suggesting some damage to intestinal tissue (Fig. 3a). In contrast, plasma levels of liver-type fatty acid-binding protein (L-FABP) were reduced in animals receiving sevoflurane narcosis (Fig. 3b), and hepatic tissue levels of IL-6 were also reduced (Fig. 3c). Levels of HMGB1 in liver tissue were unaltered by the exposure to sevoflurane (Fig. 3d).

Histological examination of hematoxylin and eosin-stained sections of both, intestinal and liver tissue did not reveal any morphological differences between control and narcosis animals (Fig. 3e & f).

### Altered kidney function

**Fig. 4:**
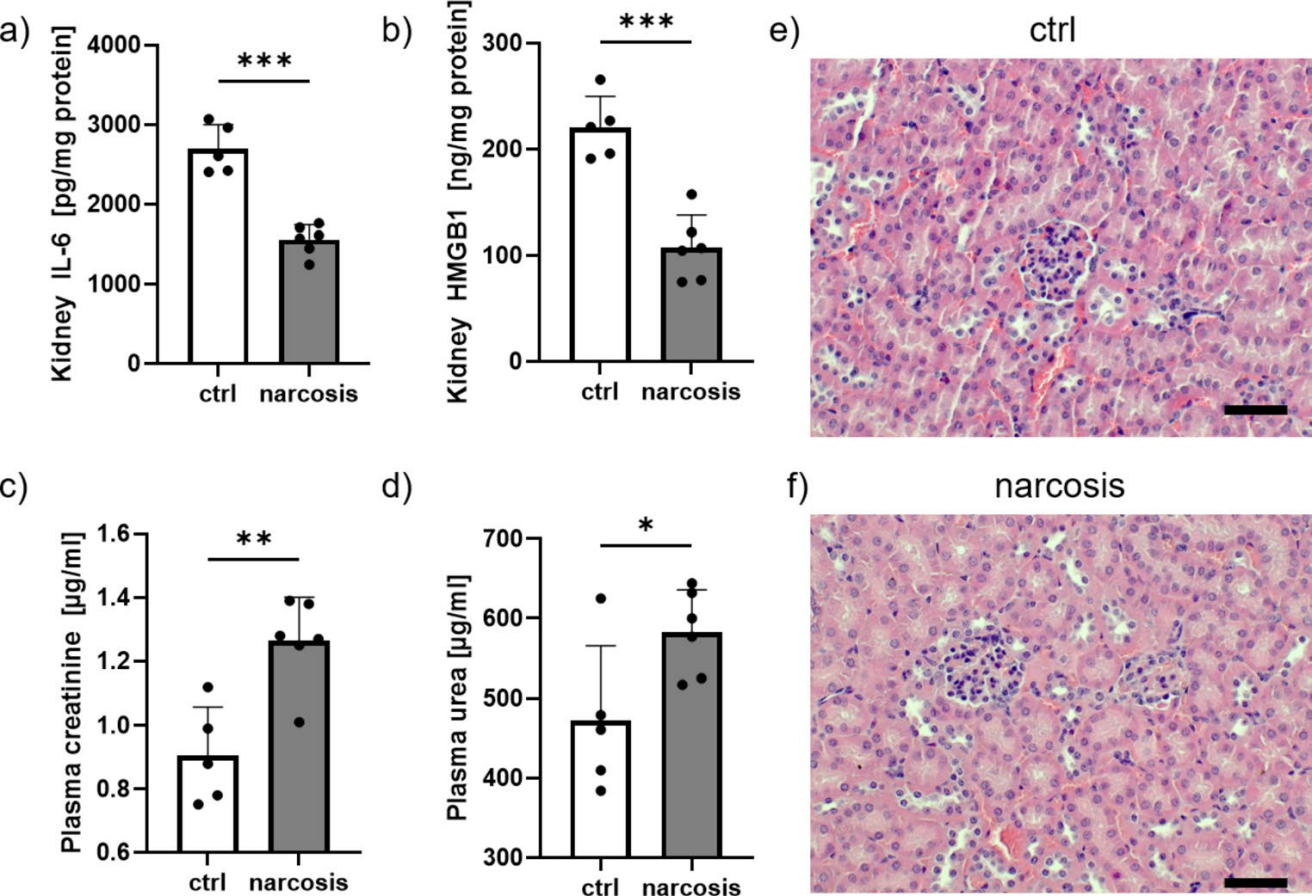
Effects of narcosis on the kidneys. Levels of interleukin 6 (IL-6, **a**) and high mobility group box protein 1 (HMGB1, **b**) were reduced in kidney tissue in the narcosis group. Plasma concentrations of creatinine (**c**) and urea (**d**) were elevated after narcosis. Histological examination did not show differences between both groups (**e**, **f**). Scale bars indicate 50 μm. *n* = 5–6 per group, *p < 0.05, **p < 0.01, ***p < 0.001, t-test, mean ± SD, no outliers

Tissue concentrations of IL-6 (Fig. 4a) and HMGB-1 (Fig. 4b) were reduced in the kidneys after prolonged anesthesia. In contrast, plasma levels of creatinine (Fig. 4c) as well as urea (Fig. 4d) were significantly elevated in the narcosis group, indicating some compromised renal function. The kidney morphology remained normal irrespective of sevoflurane exposure as assessed by histological examination of renal tissues (Fig. 4e & f).

## Discussion

Almost every experimental study in the field of trauma, shock and sepsis requires narcosis. However, not every study protocol includes control groups undergoing the same anesthesia protocol as the experimental group, but only untreated animals. But not only trauma or shock, but also anesthesia itself might alter outcome parameters. Therefore, this study aimed to investigate the influence of a standardized sevoflurane narcosis on the immunological and organ functions in a murine model. We used sevoflurane as an established volatile anesthetic in experimental as well as in clinical settings. In contrast to isoflurane, sevoflurane does not emphasize the activity of the sympathetic nervous system and thereby potentially influence outcome parameters, and is also more commonly used also in clinical settings. Nevertheless, isoflurane is still a common drug used for anesthesia in experimental settings. For this study, we chose a duration of 270 min. In many rodent models of trauma, HS and burn injury, timepoints between 2 and 6 h are used to investigate the early immunologic effects. Therefore, we decided to choose a duration in this time frame. Of course, different exposure times to volatile anesthetics might result in different emphasis of immunological changes. Furthermore, different anesthetics might have different effects on organ systems and systemic inflammation, which as a limitation are not assessed in this study.

IL-6 as a very common inflammation and outcome parameter in experimental and clinical studies concerning trauma and emergency medicine [15] was not altered by sevoflurane narcosis, with no detectable levels in either group, which is consistent with results from previous studies [16]. In contrast, other studies demonstrated an exacerbated liberation of IL-6 by narcosis, if some additional stimuli (e.g. instrumentation and mechanical ventilation or a mild LPS-bolus, respectively) were present [17, 18]. Concerning other inflammatory markers, we found an increase in KC blood levels in the narcosis group. KC represents the murine homolog of human IL-8 and acts as a potent leucocyte chemoattractant and proinflammatory mediator [19]. Therefore, increased levels of KC may represent a shift towards a proinflammatory immune response. In contrast, plasma levels of HMGB-1 as an important DAMP were reduced in mice undergoing anesthesia, rather indicating diminished tissue damage and anti-inflammation. There are virtually no other studies investigating the effects of anesthesia on KC or HMGB-1 in murine models that would allow any further comparisons. The increased levels of KC together with lowered concentrations of HMGB-1 draw a divisive picture of the systemic immune response towards narcosis, requiring further investigation. Additionally, comparisons between the few available studies are hampered by the different anesthetics used, each one having its distinct immunomodulatory properties [20].

Lungs of mice undergoing anesthesia showed significantly reduced levels of HMGB-1 and IL-6, indicating some antiinflammatory or organ protective effect of sevoflurane in lung tissue. Furthermore, this observation is reinforced by lowered PMN cell counts and significantly reduced MPO activity in the BAL fluids. Several other studies suggested protective and anti-inflammatory effects of sevoflurane on the lungs, either in the context of infection or ventilator-induced injury [21–23]. Several mechanisms through which the protective effect of sevoflurane could be mediated have been proposed, such as a reduced expression of Toll-like receptors and cytokines as well as reduced apoptosis of endothelial cells [24–26]. However, the exact signaling pathway is still unclear and needs further investigation.

Systemic L-FABP concentrations as a surrogate marker for hepatic damage were reduced in the narcosis group. There is no further mechanistic data on how sevoflurane by itself influences liver function, but some studies indicated minor protective effects of sevoflurane in models of ischemia/reperfusion injury, either of the liver itself or as remote organ during lung transplantation, by reducing inflammatory response and apoptotic processes [27, 28]. Nevertheless, there are very rare cases with fulminant fatal liver failure after sevoflurane narcosis [29, 30]. In the present study, i-FABP as an intestinal damage marker was elevated in the narcosis group, whereas morphological alterations in intestinal tissues could not be found in histological examination.

The inflammatory activity in the kidneys was reduced by sevoflurane narcosis, represented by lower concentrations of IL-6 and HMGB-1 in tissue samples, again suggesting some anti-inflammatory effects of sevoflurane. Nevertheless, renal function was impaired in mice undergoing anesthesia, represented by increased plasma levels of creatinine and urea. Of note, mice did not receive fluid supplementation during the experimental period. Therefore, reduced kidney function might also at least partly be due to dehydration. Previous studies showed an increased risk for the development of acute kidney injury (AKI) after anesthesia with sevoflurane as compared to propofol [31], and genotoxicity of sevoflurane on kidney cells [32]. In contrast, other investigations did not detect any toxicity on kidneys, or even showed protective effects of sevoflurane [33, 34]. All of these studies investigated very different preclinical and clinical settings, which makes it difficult to draw a final conclusion, especially since anesthesia-associated renal changes may add to the risk of AKI driven by trauma, shock, sepsis and other critical diseases [35].

Taken together, sevoflurane anesthesia alters some innate immune and organ functions in a corresponding murine narcosis model. Immunological and functional changes seem to be organ-specific and therefore not easy to determine. As relevant outcome parameters in trauma studies may be affected by a concomitant narcosis, all experimental studies in this field should include a control group undergoing the same anesthesia protocol as the experimental groups in order to reduce confounding factors and to prevent under- or overestimation of immunological and functional changes caused by the intervention of interest.

## Data Availability

No datasets were generated or analysed during the current study.
